# Similarities between the Effects of Prenatal Chlorpyrifos and Valproic Acid on Ultrasonic Vocalization in Infant Wistar Rats

**DOI:** 10.3390/ijerph17176376

**Published:** 2020-09-01

**Authors:** Miguel Morales-Navas, Sergio Castaño-Castaño, Cristian Pérez-Fernández, Ainhoa Sánchez-Gil, María Teresa Colomina, Xavier Leinekugel, Fernando Sánchez-Santed

**Affiliations:** 1 Department of Psychology and Health Research Center, University of Almería, Ctra. Sacramento, s/n, 04120 Almería, Spain; sergio.castano@uneatlantico.es (S.C.-C.); cpf603@ual.es (C.P.-F.); asg837@inlumine.ual.es (A.S.-G.); 2 Department of Health Sciences, Universidad Europea del Atlántico, Calle Isabel Torres, 21, 39011 Santander, Spain; 3 Department of Psychology and Research Center for Behavior Assessment (CRAMC), Universitat Rovira i Virgili, C/Carretera de Valls, s/n, 43007 Tarragona, Spain; mariateresa.colomina@urv.cat; 4 Institut de Neurobiologie de la Mediterranée (INMED), INSERM UMR1249, Aix-Marseille University, Parc Scientifique de Luminy BP.13, CEDEX 09, 13273 Marseille, France; xavier@arcadi.eu

**Keywords:** autistic spectrum disorder, ultrasonic vocalizations, chlorpyrifos, valproic acid, prenatal exposure, autism, organophosphates

## Abstract

Background: In recent years, ultrasonic vocalizations (USV) in pups has become established as a good tool for evaluating behaviors related to communication deficits and emotional states observed in autism spectrum disorder (ASD). Prenatal valproic acid (VPA) exposure leads to impairments and social behavior deficits associated with autism, with the effects of VPA being considered as a reliable animal model of ASD. Some studies also suggest that prenatal exposure to chlorpyrifos (CPF) could enhance autistic-like behaviors. Methods: In order to explore these similarities, in the present study we tested whether prenatal exposure to CPF at GD12.5–14.5 produces effects that are comparable to those produced by prenatal VPA exposure at GD12.5 in infant Wistar rats. Using Deep Squeek software, we evaluated total number of USVs, latency to the first call, mean call duration, principal frequency peak, high frequency peak, and type of calls. Results: Consistent with our hypothesis, we found that exposure to both CPF and VPA leads to a significantly smaller number of calls along with a longer latency to produce the first call. No significant effects were found for the remaining dependent variables. Conclusions: These results suggest that prenatal exposure to CPF could produce certain behaviors that are reminiscent of those observed in ASD patients.

## 1. Introduction

Vertebrates are usually able to produce acoustic signals to transmit all types of information, from characteristics related to themselves, such as identity, mood, and status, to a wide variety of environmental situations. Due to the multi-functional nature of such signals, a wide variety of different sounds can be found. In rats, such sounds are referred to as vocalizations.

Depending on their frequency and temporal properties, vocalizations can be used to communicate information on a number of dimensions, including fear, play, aggression, or proximity of a predator. In 1956, Zippelius and Schleidt built a device to detect ultrasonic vocalizations (USV; those that have a range between 20–100 kHz) from pups [1], which are, perhaps, the most interesting types of vocalizations because these are the main way in which newborns communicate with their mothers in situations of distress. Thus, USVs are a highly useful parameter for investigating neonatal behavior, particularly if we consider that pups—due to their developmental stage—are unable to express a wide range of complex behaviors. For instance, it has recently been suggested that when infant rats emit 40-kHz USV, this is because they are suffering from a very negative episode, such as deep fear and anxiety, or proximity of a predator. A USV of 66-kHz, however, is usually taken to indicate a mildly aversive situation or even a positive experience [2].

Autism spectrum disorder (ASD) is a complex neurodevelopmental disorder, which is primarily characterized by persistent deficits in social communication and social interactions, as well as restricted and repetitive patterns of behavior. For instance, autistic children show deficits in both verbal and non-verbal communication, whilst a number of studies have revealed that ASD children show abnormal patterns of crying, such as higher fundamental frequency and poor phonated production [3].

Whilst the etiopathogenesis of ASD is still unknown, it is generally accepted that this disorder is the product of multiple factors, and is likely to have a polygenic basis [4]. Nevertheless, the research on environmental factors as possible causes is also strongly supported due to the effect of exposure to certain agents during pregnancy (e.g., valproic acid or Thalidomide) [5]. The valproic acid (VPA) rat model of autism has been widely used due to the variety of structural and behavioral characteristics observed in individuals with autism [6]. VPA rats tend to show a lower preference for social interaction than comparison with controls. For instance, using a 3-chambered paradigm, it has been found that VPA rats exhibited a reduction in sociability, spending more time with an object than with a congener, whilst they did not show preference for social novelty, spending more or less the same time with a familiar rat as with a stranger [7]. Furthermore, in an open-field arena, VPA rats showed less play behavior than control rats [8]. Taken together, these findings are compatible with many of the core symptoms of ASD, i.e., those related to a lack of social communication. Using USV recordings, it has been seen how VPA pup rats exhibit fewer vocalizations, along with changes in the types of calls made in comparison with control rats. In addition, VPA rats show a higher number of simple and atypical calls compared with controls exposed to saline [9].

Among the different environmental factors that appear to be involved in the etiology of ASD, exposure to organophosphates (OPs) has emerged as a particularly strong candidate [10]. Although OPs represent an extremely large group, chlorpyrifos (CPF, diethyl 3,5,6,-tricholoro-2-pyridyl phosphorothionate), a pesticide introduced on the market in 1965 to control pests in crop fields and the houses, with a multitude of forms of employment that can be applied by aerial spraying, chemigation, spreaders drawn by agricultural machinery or hand-held equipment [11] has been the most widely used in recent years [12], in spite of the fact that in 2001 was banned by the EPA for its residential use [13]. In this regard, some reports have suggested that proximity to crop fields is a potential factor that increases the risk of autism in humans [14]. Considering this premise, and in spite of the criticism that has been directed toward the use of CPF exposed rats as an animal model of autism [15], there are some similarities between the symptoms of ASD and the effects observed in subjects prenatally exposed to CPF. These include impairments in social interactions in mice born from mothers treated with 5 mg/kg between GD12–15 [16], social communication deficits observed in pups of mothers treated with 6 mg/kg between GD14–17 [17], lack of reaction to social novelty and delayed onset of social investigation (only in female mice which came from mothers who had been given the drug orally between days GD14–15) [18], and anxiogenic behaviors in the pups of mothers treated with doses of 0.01, 0.1, 1, and 10 mg/kg/day between GD 14–20 [19].

In this study, we examined the effects of CPF administered at gestational days 12.5–15.5 (which is approximately equivalent to the late first semester of human pregnancy). In particular, we evaluated the similarities between CPF and VPA exposed animals (an accepted animal model of autism) at gestational day 12.5, comparing both of these prenatal treatment groups with a control group (CNT). On the basis of previous findings suggesting similarities between the two treatment groups, we hypothesized that VPA rats and CPF rats will show alterations in the number of USVs in comparison with group CNT. Furthermore, we also expected to find similar sound wave properties, such as peak frequency, shape, and duration.

## 2. Materials and Methods

### 2.1. Experimental Animals

The subjects were 25 pregnant Wistar rats (Janvier Labs; Le Genest-Saint-Isle, France) aged 3-months. All subjects were individually housed in our facilities in clear polycarbonate cages (50 × 15 × 24 cm). The environment was regulated (temperature: 22 ± 2 °C; humidity: 50 ± 10%, and reversal light/dark cycle with lights on from 19:00 h to 07:00 h), and the rats were acclimated to their new environment for 6 days.

The dams gave birth on the expected day. The birthday was considered as postnatal day 0 (PND0). On PND1, the pups were separated from their mothers and randomly distributed so that 10 pups were allocated to each dam (5 females and 5 males). Throughout the experimental procedure, the dams had free access to food and water.

In order to monitor signs of intoxication, the pups were weighed regularly. We began this routine on PND10 to prevent any extreme maternal reactions that could affect the pups.

This study forms part of the project PSI2017-86847-C2-1-R and was conducted in accordance with the Spanish Royal Decree 53/2013 and the European Community Directive (2010/63/EU) for animal research and approved by the University of Almeria Animal Research Committee.

### 2.2. Administration Protocols

On gestation day (GD) 11, the dams were randomly assigned to one of the following three experimental groups: Control group (CNT), chlorpyrifos group (CPF), and valproic acid group (VPA). On GD12.5, all dams began the treatment according to the following protocols:

- CNT (n = 8): One subcutaneous injection of 1 mL/kg of dimethyl sulfoxide (DMSO) for four days.
- CPF (n = 8): One subcutaneous injection of 1 mg/kg of CPF [O, O-dietil O-3,5,6-trichloropyridin-2-yl phosphorothioate (Pestanal, Sigma Aldrich)] dissolved in DMSO, for four days.
- VPA (n = 9): One subcutaneous injection of 400 mg/kg body weight of VPA (with the aim of avoiding possible maternal death (Kim et al., 2011)), dissolved in 0.9% saline at a concentration of 250 mg/mL; and three daily subcutaneous injections with saline only.

In order to ensure that correct dosages were administered, the rats were weighed daily. The schedule was completed on GD15.5, when all dams had received four subcutaneous injections each (Table 1).

### 2.3. Ultrasonic Vocalizations (PND7)

Ultrasonic vocalizations were recorded using an ultrasonic microphone (Dodotronic ultramic 250K, Dodoctronic di Ivano Pelicella, Castel Gandolfo, Italy) within a sound-attenuating chamber (80 × 60 × 70 cm) positioned at a height of approximately 10 cm above the animal [20]. The software used to conduct and analyze the recordings was SeaWave software version 2.0 (CIBRA), with a sampling rate of 250 kHz, in a 16-bit format. The temperatures were regulated inside the boxes, and in the adjacent room and experimental room (22 ± 2 °C).

First, all experimental cages were placed in a room adjacent to the experimental room (both with the same levels of humidity and light conditions as the home room) for 20 min for acclimatization. The isolation procedure was then applied, which was the same as that employed by Hofer, Shair, and Brunelli [21], with one exception. With the aim of avoiding any effects of maternal stress on the pups prior to the test, we placed them in a polycarbonate box with a thermal blanket underneath. Once they had been placed inside this box, the pups were covered with cotton from the nest, as well as some sawdust and droppings collected from their home cage, with the purpose of providing them with the familiar features of their home environment in order to avoid the potential effects of a contextual change during the test. Each pup was then carefully taken and placed inside the soundproof box for 3 min (following the interval of 3–6 min suggested by Hofer, Shair, and Brunelli [21] to obtain good recordings) during which each vocalization was recorded. After each session, the box was cleaned with 70% ethanol. All the recordings were conducted from 08:00 h to 13:00 h.

In total, the recordings of 42 litters PND7 (Males: 7 CNT, 7 CPF, and 7 VPA; Females: 7 CNT, 7 CPF, and 7 VPA) were analyzed. All recordings were selected randomly until each experimental group was completed. We chose PND 7 because it seems to be the day where the optimal peak is found in which the most calls occur [21,22,23].

The parameters analyzed were the following: Total Number of Calls, Latency to the First Call, Mean Call Duration, Principal Frequency Peak, High Frequency Peak, and Call Classification. The latter was based on Wright’s classification [24]. The software used to obtain these parameters was DeepSqueak [25] which uses regional convolutional neural networks (Faster-RCNN) to conduct the analyses [26]. In order to ensure experimental rigor and with the purpose of not losing the data from any call, we conducted the analysis twice with two different methods of Neural Network detection: All Short Calls_Network_V1 and Long Rat Call Network_V2; since both of these methods are included by default, which means the following: Analysis Chunk Length 6, Overlap (seconds) of 0, 1, High Frequency Cut-Off (kHz) of 100, Low Frequency Cut-Off (kHz) of 18; and Score Threshold (0-1) of 0. The computer used to carry out the analyses was an MSI GT72VR 7RE Dominator Pro with a CPU Intel Core i7-7700HQ and a Nvidia GTX1070 as Graphic Card. After the detection phase, all recordings were manually checked with the intention of correcting possible errors and rejecting noise.

### 2.4. Acetylcholinesterase Activity

At PND1, the pups (10 CNT, 4 CPF, 7 VPA) were sacrificed by cervical dislocation and the whole brain was quickly removed, flash-frozen and stored at −80 °C. In order to analyze acetylcholinesterase activity, the brains were thawed and homogenized with Triton X-100 in 0.1 Na phosphate buffer (pH 8) at a ratio of 1/10 (*w/v*). Samples were then sedimented at 15,000× *g* for 15 min, and the aliquots of the supernatant were diluted with 0.1 M Na phosphate buffer (pH 8) at a ratio of 1/10 (*w/v*). We followed a slightly modified version of Ellman’s Method [27], using 10 µL of this dilution (all determinations in duplicates) mixed with 5,5-dithiobis-2-nitrobenzoic acid (DTNB) (60 µL; final concentration = 0.33 nM) and 206 µL of sodium phosphate buffer (0.1 M; pH 8.0). The mixture was homogenized for 30 s at room temperature and incubated for 5 min at 37 °C. Following this step, we added 15 uL of the butyrylcholinesterase blocker tetra isopropyl pyrophosphoramide (final concentration = 0.5 nM). As a substrate, we used 9 µL of acetylthiocholine iodide (diluted in 0.1 M Na phosphate buffer; pH 8.0; final concentration = 0.5 mM). The absence of acetylthiocholine in the blanks was completed with an extra sodium phosphate buffer. Using a spectrometer, we measured the rate of acetylcholine iodide hydrolysis at 412 nm for 22 min at 37 °C.

### 2.5. Statistical Analysis

A two-way ANOVA was conducted to analyze total number of calls, latency to the first call, call duration, principal frequency, and high frequency peak. The two factors were Sex (Male and Female), and Treatment (CNT, CPF, and VPA). Post-hoc analysis was conducted using the Bonferroni test. The number of calls recorded throughout the entire session, and the weights of the pups throughout the course of the experiment were analyzed using a repeated- measures ANOVA.

The probability of vocalization was analyzed through an ANOVA for each type of vocalization, standardized by angular transformation for each subject/total number of calls.

To calculate acetylcholinesterase activity, we selected a 1-min sample of the enzymatic reaction, which was used for subsequent analysis using the Beer–Lambert law, after which this was corrected according to the total amount of protein analyzed following the Bradford protein assay. We then conducted a univariate test with treatment as the independent variable.

Bootstrapping was applied in those cases in which the Levene Test was significant in order to conduct the pairwise comparisons with more confidence. We adopted a significance level of *p* < 0.05 for all analyses. The results are expressed as means ± SEM. All the analyses were conducted using IBM SPSS (version 22.0.0.0; Chicago, IL, USA), and the graphs were created with GraphPad Prism version 6.0 (San Diego, CA, USA).

## 3. Results

### 3.1. Developmental Markers

**Figure 1:**
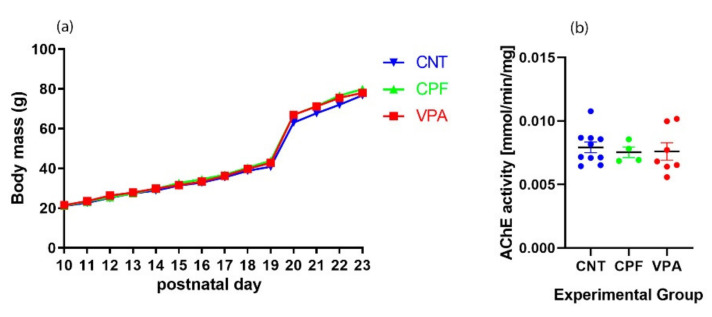
(**a**) Body weight gain as a function of days. There were no significant differences in weight evolution in relation to the different prenatal treatments received by each group; (**b**) Effects of prenatal treatment on the expression of AChE. There were no significant differences between the groups. The data are expressed as a ratio of concentration of average control ± SEM. Each dot or square represents an individual animal (CNT = 10; CPF = 4; VPA = 7).

No significant differences in body weight were found between the groups (Figure 1a). Normal progression of the weights continued over time, even after weaning. Further, in comparison with the CNT group, we did not find signs of acetylcholinesterase inhibition in any of the experimental groups (Figure 1b).

### 3.2. Ultrasonic Vocalization Recordings

**Figure 2:**
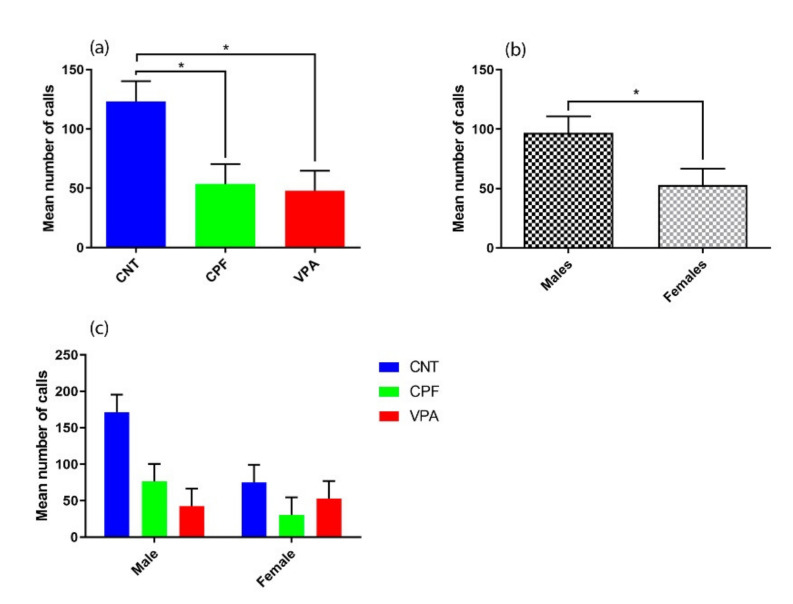
Differences in total number of calls (n = 42): (**a**) There were significant differences according to the prenatal treatment received, with control group (CNT) (n = 14) being significantly different from the other two groups: Chlorpyrifos (CPF) (n = 14) and valproic acid (VPA) (n = 14); (**b**) There was a significant difference between males (n = 21) and females (n = 21); (**c**) There was no significant Treatment * Sex interaction, in spite of the fact that the males of the CNT (n = 7) group tend to emit considerably more calls than any other group (CNT female n = 7; CPF male n = 7; CPF female n = 7; VPA male n = 7; VPA female n = 7), showing more clearly the how the prenatal treatments differentially affect males and females.

For the total number of calls, we found main effects of Treatment [F (2, 36) = 6.120, *p* = 0.005] (Figure 2a) and Sex [F (1, 36) = 5.007, *p* = 0.032] (Figure 2b). Post-hoc analyses revealed that the CNT group emitted more calls than both the CPF (*p* = 0.019) and VPA (*p* = 0.01) groups, with no differences observed between the CPF and VPA groups (Figure 2a). Regarding Treatment*Sex there was no significant difference between groups, and only a tendency to emit considerably more calls was observed in CNT males.

**Figure 3:**
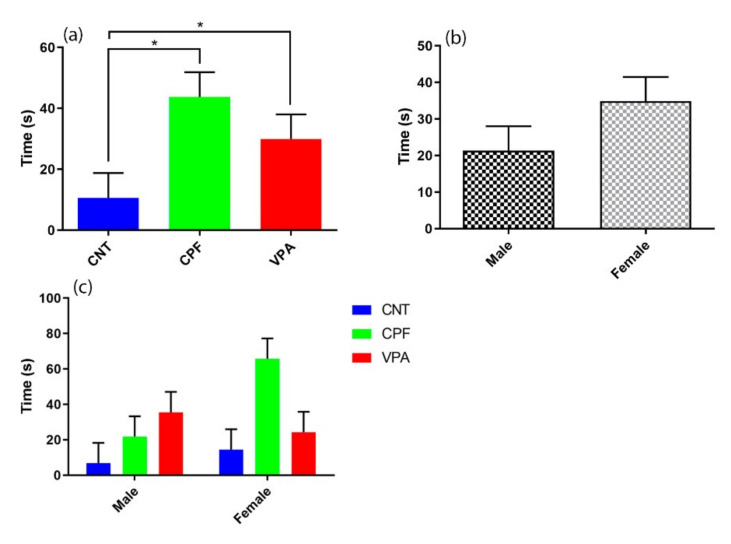
Latency to the first call (n = 42): (**a**) There were differences according to treatment, with both CPF (n = 14) and VPA (n = 14) groups differing significantly from CNT (n = 14). (**b**) There were no significant differences according to sex (male n = 21; female n = 21). (**c**) The Treatment * Sex interaction was not significant, although there is a strong tendency for CPF females to take more time to emit their first call in comparison with the other groups (CNT male n = 7; CNT female n = 7; CPF male n = 7; CPF female n = 7; VPA male n = 7; VPA female n = 7).

Furthermore, analysis of the latencies to the first call also revealed a main effect of group (F (2, 36) = 4.201, *p* = 0.023) (Figure 3a). Post-hoc analyses revealed that subjects in both the CPF and VPA groups took more time to emit the first call than group CNT (*p* = 0.013 and *p* = 0.042; respectively). No significant differences were found according for Sex or the Treatment × Sex interaction (Figure 3b,c), although the females tend to show higher latencies to the first vocalization (Figure 3b), a trend that appears to be driven by the females of the CPF group (Figure 3c).

**Figure 4:**
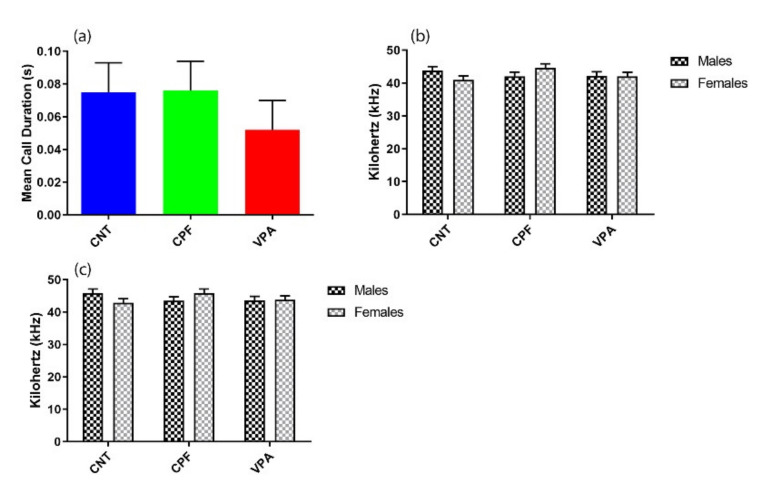
Characteristics of the USVs (n = 42): (**a**) There were no significant differences in Mean Call Duration between the treatment groups, although there is a tendency for the VPA (n = 14) group to emit shorter calls than the other two groups (CNT n = 14; CPF n = 14). Moreover, the groups did not differ in terms of either Principal Peak Frequency (**b**) or High Peak Frequency (**c**).

**Figure A1:**
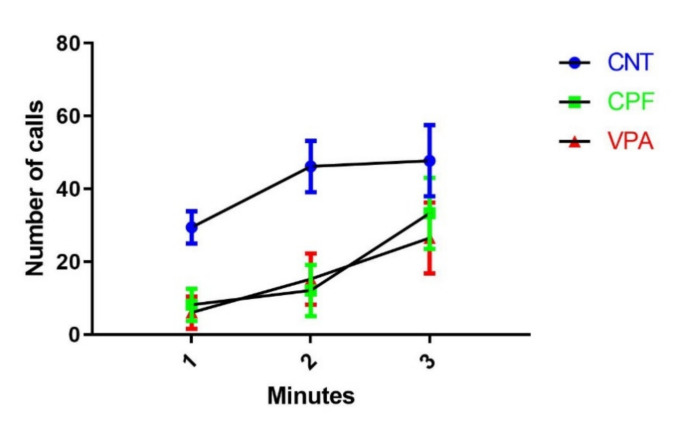
Evolution in the number of calls (CNT n = 14; CPF n = 14; VPA n = 14).

There were no significant differences between the groups for Mean Call Duration, Principal Peak Frequency, or High Peak Frequency (Figure 4a–c). Nonetheless, it appears that the duration of the calls of the VPA groups tend to be shorter than those of the other two groups. A repeated-measures analysis conducted to evaluate the progression of the calls throughout the 3 min failed to find any significant effects (Figure A1).

## 4. Discussion

Using rat subjects, the present study explored the effects on ultrasonic vocalization following prenatal treatment with a subclinical dose of CPF, in a period equivalent to the first trimester of human gestation [28]. These effects were also compared with those produced by rats prenatally treated with VPA. To the best of our knowledge, this study is the first to report the existence of similarities between the effects of prenatal treatment with CPF and VPA.

Infant body weight could be affected by both CPF and VPA. For instance, prenatal exposure to high doses of CPF can provoke a significant loss of body weight in infant rats [16,29]. In order to detect physical signs of intoxication (even though we used a subclinical dose of CPF), we weighed the pups daily and analyzed AChE activity at PND1. Neither of these measures revealed signs of toxicity in either the CPF exposed group or the other two groups, suggesting that the observed effects are independent of AChE inhibition, which is the principal mechanism of action of CPF [19]. It is also known that prenatal treatment with VPA could be the cause of lower body weight in infant rats [8], but, again, and possibly because we used a low dose (400 mg/kg), we did not find weight differences between our VPA and CNT groups.

In order to evaluate our hypothesis, it was important to establish whether the significant differences found for the positive control VPA in the existing literature were similar to the results obtained in this study, and, as we can see, we have obtained results consistent with those of other published studies regarding two of the variables: latency to the first call, and number of calls [30,31,32]. Taking this into account, and examining the data obtained from the group prenatally exposed to CPF, which showed similar results to VPA regarding the same two variables, we can assume that our hypothesis has at least been partially supported. As we have already shown subjects in the CPF group show higher latencies to the first call than the CNT group, a finding that was previously reported by Venerosi et al. [17]. However, it is also the case that in the latter study there were no differences in the number of calls between these two groups on PND7. However, this discrepancy could be due to several methodological differences between the latter study and the present study, such as: The class of rodent (they used mice and we have used rats), the routes and windows of administration (they administered the substances through intraoral gavages on GD14–17, whilst our administrations were carried out using subcutaneous injections on GD12.5–15.5), and the amount of CPF (they used 6 mg/kg, whilst we used a dose of 1 mg/kg).

Interestingly, our results revealed a pattern of sex-dimorphism in the total number of calls, with the males emitting more calls than the females. In particular, the males of the CNT group emitted more calls than the males in the other two groups. No differences, however, were found between the females of the two groups in females. These findings could be related to the fact that autism is a condition that predominantly affects males.

Given the lack of effects observed in acetylcholinesterase activity, we can rule out the possibility that toxic clinical effects were produced by the dose used in the present study. Nonetheless, and as a limitation of this study, we are aware that a Functional Observation Battery would offer more detailed evidence of any developmental deficits produced by the various treatments. Although USV analyses could entail some subjectivity problems that could hinder reliable classification [33], the number of parameters evaluated in this study has enabled us to identify those variables that are most sensitive to VPA and CPF exposure. Factors such as the movement of the pup heads, slight differences between the same kind of call, or graphic resolution of the calls [21] could increase the variability of certain parameters related to USV, thus hindering its usefulness for detecting subtle differences between groups.

Whilst there are some studies with mice that have yielded a similar pattern of results to those reported here [16,34], to the best of our knowledge, this is the first study to address the possible similarities between the effects of prenatal exposure to CPF and VPA in rats. Nonetheless, the assumption that CPF exposure could provoke some effects that are comparable with the signs observed in ASD patients should must be treated with caution. It is important to bear in mind that there are significant differences in the USVs between mice and rats [21,35], and even among different strains of rats [36,37,38] which makes it difficult to extrapolate some of the findings.

Pregnancy is possibly the most important stage in the development of a human being. Minimal changes during pregnancy can trigger very serious problems that can affect the person throughout the life span. The results of the present study suggest that neonatal CPF exposure could represent an environmental factor that potentially increases the risk of observing such problems (in this case, those related to ASD). Indeed, current epidemiological data indicate that among the general population, the incidence of ASD is 1 in 152 children [39], but in regions where CPF is use as a pesticide, the risk of suffering from this disorder increases by up to 60% [40]. Besides, in the published literature regarding prenatal administration, similarities can be found in some of the effects of both substances, such as increased oxidative stress [41,42], and alterations in serotonergic connections [43,44]; among others. Therefore, whilst further research into this issue is needed, CPF should clearly be taken seriously as a possible risk factor for the development of ASD.

## 5. Conclusions

The present study reports that prenatal exposure to CPF could cause effects similar to those seen in VPA regarding pups’ vocalizations. The amount of calls emitted by the CPF and VPA groups throughout the test are practically identical, and much less than the calls those produced by the CNT group. Furthermore, in the latency to the first call, again both groups CPF and VPA stay longer without emitting any calls.

We did not find any relevant differences in the rest of the variables related to the characteristics of the ultrasonic waves. This empirical information directly links the gestational exposure of a common pesticide to ASD-like behaviors in rats during neonatal periods, complementing previous data found in mouse-models. All things considered, the effects of CPF during the development must be further investigated even in those doses which are below the toxic threshold.

## Figures and Tables

**Table 1:** Schedule of drug administration.

Group	PND12.5	PND13.5	PND14.5	PND15.5
CNT	DMSO	DMSO	DMSO	DMSO
CPF	CPF-Drug	CPF-Drug	CPF-Drug	CPF-Drug
VPA	VPA-Drug	Saline	Saline	Saline
